# Impact of 3 months of detraining after high intensity exercise on menopause-related symptoms in early postmenopausal women – results of the randomized controlled actlife project

**DOI:** 10.3389/fspor.2022.1039754

**Published:** 2023-01-05

**Authors:** Sophia Jungmann, Michael Hettchen, Matthias Kohl, Wolfgang Kemmler

**Affiliations:** ^1^ Institute of Medical Physics, Friedrich-Alexander University Erlangen-Nürnberg, Erlangen, Germany; ^2^ Department of Medical and Life Sciences, University of Furtwangen, Schwenningen, Germany; ^3^ Institute of Radiology, University-Hospital Erlangen, Erlangen, Germany

**Keywords:** high-intensity exercise, menopause, postmenopausal women, quality of life, detraining effect

## Abstract

Regular exercise might reduce postmenopausal symptoms, however even short-moderate periods of absence from exercise training might significantly reduce these positive effects. The aim of the study was thus to determine detraining effects on postmenopausal symptoms after a 3-month detraining period in early post-menopausal women. After 13 months, the exercise group (EG: *n* = 27; 54.6 ± 2.0; 23.6 ± 3.3 kg/m^2^) had to abruptly stop their supervised, facility-based, high intensity aerobic and resistance group exercise conducted three times per week due to the COVID-19 pandemic and the corresponding lockdown of all training facilities in Germany. In parallel, the control group (CG: *n* = 27; 55.6 ± 1.6 years, 25.2 ± 5.2 kg/m^2^) had to terminate their low-intensity exercise program performed once per week. Study endpoint as determined after 3 months of detraining was menopausal symptoms as determined by the Menopausal Rating Scale II (MRS II). The intention to treat principle with multiple imputation was applied. After 13 months of intense multicomponent exercise and significant exercise-induced effects on menopausal symptoms, a further 3 months of detraining resulted in non-significant deteriorations (*p* = .106) in the exercise group, while non-significant improvements were observed in the control group (*p* = .180). Corresponding group differences were significant (*p* = .036) after detraining. Of importance, self-reported individual outdoor activities increased by about 40% in both groups during the three-month lock-down period. Three months of absence from a supervised high-intensity group exercise protocol resulted in detraining effects on postmenopausal symptoms even when outdoor physical activity was increased significantly.

**Trial registration number:**

ClinicalTrials.gov: NCT03959995

## 1. Introduction

Exercise is an effective method for easily and non-invasively improving the quality of life in middle-aged women [1]. Postmenopausal symptoms, such as hot flushes, that occur due to the menopausal transition in women are also readily alleviated by a simple sports program [2–4]. However, various life circumstances might lead to longer breaks in women's exercise habits [5] Although a few studies [6–8] focus on detraining effects on muscle strength or bone mineral density after exercise periods in women, these did not include studies focusing on menopausal symptoms. Most closely to this context, Bocalini et al. [9] reported that after 6 weeks of detraining from a 12-week water-based exercise program, the positive effects of exercise on quality of life including pain, psychological, social aspects and sexuality, were lost.

In the ACTLife study, we could confirm that a 13-month supervised high intensity exercise program triggered significant positive effects on menopausal symptoms in postmenopausal women [10]. However, due to the Covid 19 (Corona Virus Disease 2019) pandemic all training facilities had to be closed during the three-month lock down in Bavaria, Germany. These circumstances provided a good opportunity to examine the effect of a short-term detraining phase on the previously improved menopausal symptoms from the exercise program in women.

Thus, the present study aims to determine the effects of a 3-month detraining phase immediately after a prior 13-month high intensity exercise period on menopausal symptoms and complaints. Our hypothesis was that the significant exercise-induced effect on menopausal symptoms observed after 13 months of exercise was significantly reduced after a 3-month period of detraining.

## 2. Methods

This work is part of the ACTLife project, a European Project that focuses on the development and dissemination of best practice exercise protocols for therapy and prevention of osteoporosis, physical fitness, and menopausal reliefs. The present article focus on detraining effects on menopausal symptoms after the 13 month training period of the project.

The FAU Ethics Committee approved (number 118_18b) the follow-up assessment for 3 months of detraining, after the informed consent of all study participants and study registration (ClinicalTrials.gov: NCT04420806). The assessment was conducted in mid-June 2020.

### 2.1. Participants

**Figure 1:**
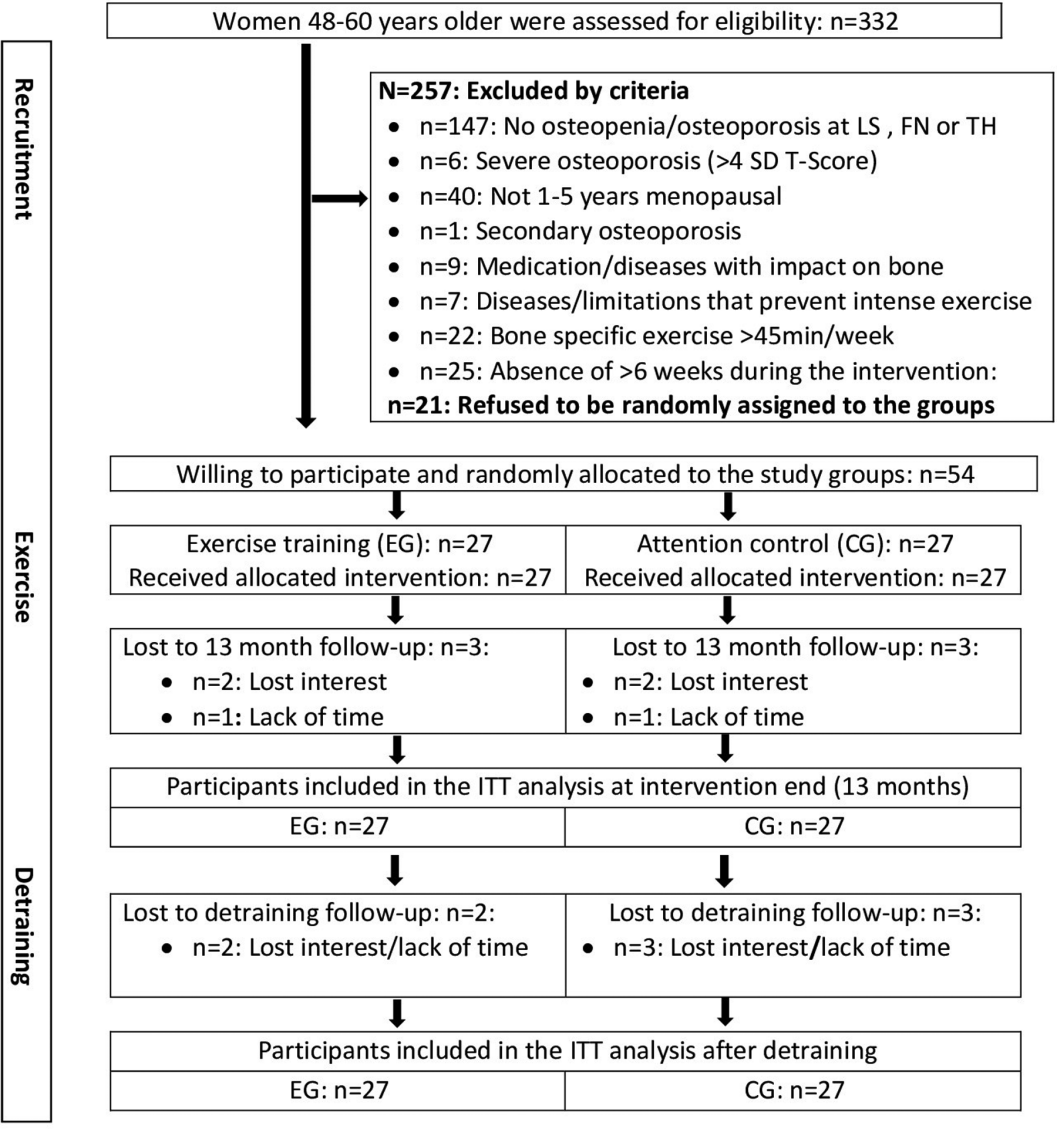
Flow chart of the study dedicated to participants with assessment of the MRS II questionnaire after intervention and detraining period.

The ACTLife -RCT recruitment process has already been described in detail [10]. Briefly, 332 women interested in participating in the studies were assessed for eligibility (Figure 1). Participants were eligible if they fulfilled the criteria displayed in figure one and agreed to participate independently of the result of the randomization procedure. Following these criteria 54 women willing to participate were randomly allocated to the study groups (Figure 1).

### 2.2. Randomization procedures

Stratified for baseline lumbar spine bone mineral density (BMD) participants were randomly allocated to the exercise (*n* = 27) and control group (*n* = 27) by a researcher not involved in the present study. Neither the researchers nor participants knew the allocation in advance (“allocation concealment”).

### 2.3. Blinding

The blinding strategy included outcome assessors and test assistants who were unaware of the participants' group status (EG or CG).

### 2.4. Study procedure

In addition to the exercise intervention, all the participants were provided with cholecalciferol (Vit-D) and calcium (Ca) supplements (up to 800 IU/d Vit-D; 1,000 mg/d Ca) [11]. The participants were requested to maintain their usual exercise habits and physical activity including their habitual dietary intake and lifestyle during the study, apart from the exercise intervention program.

### 2.5. Intervention

#### 2.5.1. Exercise group

The exercise protocol of the EG has been described in detail [10]. Briefly, we applied a multimodal approach, focusing on musculoskeletal parameters, using high impact aerobic dance jumps with moderate to high ground reaction forces and periodized high effort/high intensity (60%–85% 1RM) resistance training (HIT-RT) [12]. Three continuously supervised group sessions per week were prescribed during the 8-12-week high-intensity training phases. These were intermitted by 4-5 weeks low intensity regeneration periods. Attendance rate during the training period was 79 ± 12% in the EG.

#### 2.5.2. Control group

In the control group the exercise intervention focused on flexibility, stability, and well-being, with an exercise protocol that is unlikely to affect “maximum strength/power”, “body composition” or “menopausal symptoms” relevantly. Two periods of 12 weeks with one session of 45 min/per week of continuously supervised group exercises interspersed with 12 and 14 weeks of unsupervised, video-guided exercise at home (15 min) were performed. Attendance rate during the training period was 78 ± 14% in the CG.

### 2.6. Study outcome

• Changes of Menopausal Symptoms determined by the MRS II questionnaire [13] from intervention end (13 months) to 3-month detraining follow-up.

### 2.7. Assessments

Standardized assessments and tests were consistantly used. Participants were asked to avoid intense physical activity and changes of dietary intake 48 h before the tests. The test was performed at about the same time of day (±90 min), with the same calibrated equipment and/or the same protocols and specifications.

Menopausal symptoms (hot flushes, heart discomfort, sleep problems, depressive mode, irritability, anxiety, physical and mental exhaustion, sexual problems, bladder problems, dryness of vagina, joint and muscular discomfort) were determined by the MRS II questionnaire (Menopause Rating Scale II) [13].

A standardized basic questionnaire has to be completed by all participants and asked about (a) demographic parameters; (b) diseases, physical limitations, and pharmacologic therapy with special emphasis on the risk of osteoporosis and ability to frequently conduct intensive exercise; (c) dietary supplements; (d) frequency and severity of lumbar spine pain and (e) lifestyle, including physical activity and exercise. The follow-up (FU) questionnaires focused on changes in parameters (i.e., pharmacologic therapy, diseases, surgery, lifestyle, diet, exercise) that might have influenced the present study results. The questionnaires were then reviewed together with the participants. Great importance was attached to completeness, accuracy, and consistency.

At study start (baseline) and after 7, 13 and 16 months, dietary intake was recorded on three weekdays and a weekend day characteristic for dietary habits. Participants were given simple dietary protocols (Freiburger Nutrition Record, nutri-science, Hausach, Germany), which were consistently analyzed by the same researcher.

### 2.8. Statistical analysis

We carried out intention to treat (ITT) analysis which included all participants originally assigned to the EG and CG. Using R statistics software ITT was performed in combination with Amelia II (a program for missing data). The complete data set was used for several imputations. The imputation was repeated 100 times. With respect to the MRS II-analysis, six missing 13 month-FU (i.e baseline date of the present study) datasets and five missing 16 month-FU (after detraining) datasets (Figure 1) were imputed. Imputation worked well. The normal distribution of the study endpoints was checked by graphical (qq-plots) and statistical (Shapiro-Wilks) procedures. We have applied an ANCOVA adjusted for 13-month data (i.e., baseline data of the present study) to compare changes for the detraining period between the EG and the CG. Changes over time within the groups were investigated by paired t-tests using the Barnard and Rubin approach [14]. 2-tailed tests were applied, and significance was accepted at *p* < 0.05.

## 3. Results

The 13-month characteristics of the EG and the CG, which can be considered as the baseline data of the present project, are displayed in Table 1. Participant characteristics were comparably distributed, however body mass varied considerably but non-significantly. In contrast to dietary calcium intake, dietary protein intake was very high in both groups.

**Table 1:** 13-month follow-up characteristics of the ACTLife-study. Data based on imputation of missing values (CG: *n* = 7, EG: *n* = 6) by Amelia II.

Variable	CG (*n* = 27) MV ± SD	EG (*n* = 27) MV ± SD	*p*
Age (years)	55.6 ± 1.6	54.6 ± 2.0	.441
Years after menopause (years)	4.5 ± 1.1	4.8 ± 1.0	.838
Body height (cm)	164.5 ± 8.2	164.2 ± 6.0	.889
Body mass (kg)	68.4 ± 14.1	63.7 ± 10.2	.116
Total body fat (%)	33.9 ± 6.9	32.3 ± 5.3	.414
BMD at Lumbar Spine (g/cm^2^)^a^	.895 ± .111	.875 ± .119	.571
BMD at total Hip (g/cm^2^)^a^	.815 ± .081	.803 ± .107	.687
Energy intake (kcal/d)^b^	2088 ± 387	2051 ± 403	.925
Protein intake (g/kg/body mass/d)^b^	1.16 ± 0.19	1.21 ± 0.23	.361
Individual outdoor activity (min/week)	119 ± 90	126 ± 88	.771

^a^ As determined by DXA (Hologic QDR 4,500 Discovery-upgrade, Hologic Inc., Bedford, USA).

^b^ As determined by a 4-day dietary protocol.

*Please add “Table 1: 13-month Follow-up characteristics of the ACTLife-RCT study” about here*.

In summary, three women in the EG and three women in the CG group quit the study during the intervention period (Figure 1). A further two and three participants of the EG and CG respectively were lost to follow-up during the detraining period (Figure 1). As stated, missing MRS II data of these participants were imputed by Amelia II.

### 3.1. Study outcomes

At study start all the women reported menopausal symptoms. Of importance for the subsequent detraining results, menopausal symptoms as determined by the MRS II (Menopausal Rating Scale) changed favorably in both groups, although the changes after 13 months of intervention were only significant in the EG (EG: *p* = 0.002; CG: *p* = 0.891). Corresponding between group-differences after the exercise intervention were significant (*p* = .029). In detail, based on similar baseline values, changes in all subscales of the MRS II, i.e., the somato-vegetative (*p* = .013), psychological (*p* = .123), and urogenital complex (*p* = .202), were more favorable in the EG compared to the CG.

**Figure 2:**
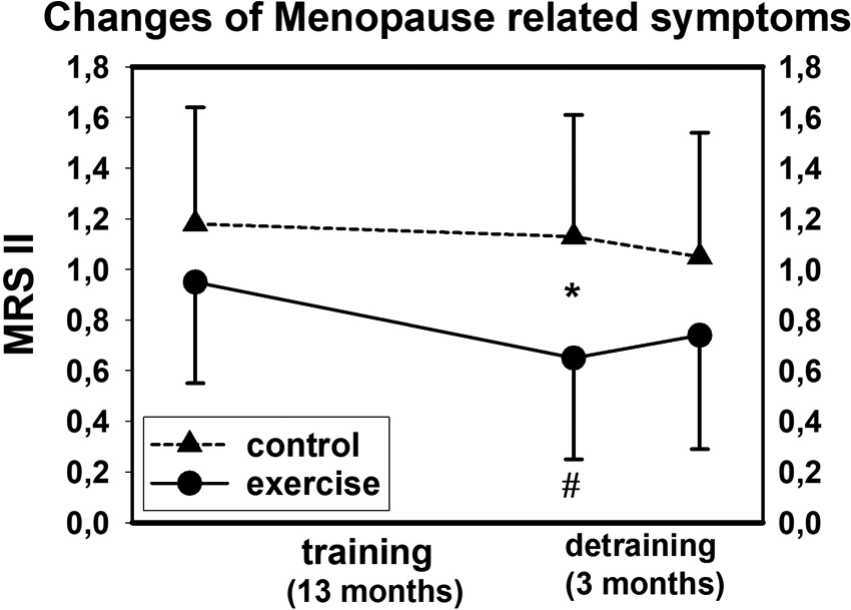
Mean values and SD for changes of menopausal symptoms (MRS II) after training and detraining. # *p* < .05 for within group differences, *p < .05 for between group differences.

With respect to our primary research question, as hypothesized, the significant exercise effect on menopausal symptoms described above was lost after a further 3 months of detraining. In detail, we observed non-significant deteriorations in the EG (*p* = .106) and non-significant improvements in the CG (*p* = .180), resulting in significant group differences and corresponding detraining effect (*p* = .036) between the groups (Figure 2). Changes in all domains (somato-vegetative, psychological, and urogenital subscales) were non-significantly positive in the CG (*p* ≥ .118) and non-significantly unfavorable in the EG (*p* ≥ .088). The group difference for the somato-vegetative subscale was significant (*p* = .044), while the detraining effect on the psychological (*p* = .071), and urogenital complex (*p* = .104) was less pronounced and failed to reach significance.

Please add “Figure 2: Mean values and SD for changes of Menopausal Symptoms (MRS II) after training and detraining. # p < .05 for within group differences,* p < .05 for between group differences” about here.

### 3.2. Confounding parameters

There were no relevant changes or group differences of dietary intake, disease, or pharmacological therapy during the detraining period. However, the volume (min/week) of individual aerobic outdoor activities such as brisk walking, jogging, or cycling increased significantly (*p* < 0.001) in both groups (EG: 41% vs. CG: 37%; *p* = .881). Further, all but four participants of the EG and CG each conducted the 15-min exercise video session at least once per week (EG: 1.4 ± 0.7 vs. CG: 1.7 ± 1.0; *p* = .586) during the detraining period.

## 4. Discussion

Little is known about the effect of detraining on the menopausal transition. While many studies address detraining effects on quality of life [9], metabolic profile, body composition [15, 16], muscle strength/mass [6, 15, 17] or bone mineral density [7, 15] in perimenopausal or (early) postmenopausal women, none investigated the effect of detraining on menopausal symptoms. This article provides the first published data of detraining effects on menopausal symptoms in early-postmenopausal women. The present findings are particularly important for women who struggle with menopausal symptoms [18–20], but cannot maintain regular exercise [5].

Summarizing the study findings, the exercise-induced significant effect on menopausal symptoms was lost after 3 months of detraining (*p* = .036). Changes in the MRS II score and all underlying domains (somato-vegetative, psychological, and urogenital subscales) were favorable for the CG and negative for the EG, with significant group differences for the somato-vegetative subscale (*p* = .044). Due to the lack of comparable studies, it is difficult to rate the clinical significance of our finding. There is some evidence that an intervention effect of 0.3–0.5 (ACTLife: 0.31) on the MRS II scale can be considered clinically significant, however we doubt that this finding can be applied to our finding on detraining effects addressed here.

Reviewing the literature, although menopause symptoms were not investigated, Bocalini et al. [9] reported a loss of favorable exercise effects on quality of life in older women after a 12-week water-based exercise program and 6 weeks of subsequent detraining. Ockene et al. [20] and Lindh-Astrand et al. [21], described recurring or persisting vasomotor symptoms after the cessation of hormone replacement therapy (HRT) in postmenopausal women. Although exercise-induced changes of estradiol (E2)-levels [22] were considerably lower compared to HRT-induced changes, the cessation of high intensity exercise and corresponding reductions of E2-levels could provide a contribution to the physiological explanation of our findings. Further, increases in ß-endorphins triggered by intense exercise significantly reduced LH and FSH levels in women with vasomotor symptoms [23]. Consequently, lower LH and FSH might result in lower vasomotor symptoms during menopause [23]. This interaction could be an explanation for the significant effects of training and detraining on somato-vegetative symptoms observed in the present study.

Of note, in contrast to the decrease in menopausal symptoms in the CG potentially as a usual effect over time [24, 25], the MRS II score increased (non-significantly) in the EG during the detraining period. Thus, the normal process of decreases of menopausal symptoms over time might be overwhelmed by the pronounced negative effect triggered by the rapid cessation of the training program. With respect to the high variance of the individual changes (Figure 2), one may argue that sociodemographic characteristics, lifestyle, and health problems known to affect menopausal symptoms [24–26] have confounded our finding. However, the lack of relevant changes in sociodemographic, lifestyle, and health characteristics during the detraining period did not support this speculation.

It should be mentioned that the detraining approach of ACTLife was not based on a preplanned study design. In fact, most of the limitations of the ACTLife detraining project are related to the rapid and unintended study termination of the ACTLife intervention period. This might be most clearly indicated by the lack of a dedicated sample size analysis for detraining effects on the MRS II scale in this study. Nevertheless, the pandemic allows us to concentrate on a research topic that we would have otherwise hardly addressed. While we consider a preplanned training break to generate detraining effects morally dubious, the COVID-19-induced cessation of the ACTLife intervention allows us to determine the effects of detraining on postmenopausal symptoms. Another feature, the mandatory closure of all training facilities in March 2019, also meant that none of the women were able to continue supervised high intensity exercise training during the 3-month detraining phase. However, it must be emphasized that both groups similarly increased (*p* < .001) their individual outdoor aerobic activities and comparably applied the video-guided 15 min home exercise protocol during the detraining period.

One may also argue that the detraining effects were predominately related to the situation of the COVID 19-related lockdown. There is some evidence that the severe COVID-19-induced change in daily routines influenced psychosocial effects related to MRS II domains in this cohort of early-postmenopausal women [27]. However, this applies to both the EG and CG alike and thus does not relevantly affect the reliability of our results. Of further importance, ACTLife focused on osteopenic, early-postmenopausal women (12–60 months of amenorrhea). While we are not aware of studies that reported differences in menopausal symptoms (or their changes) in women with or without osteopenia/osteoporosis, the study approach to exclude the relevant cohort of perimenopausal women who are particularly affected by menopausal symptoms decreases the generalization of our findings considerably.

In conclusion, the present study provided further insights into detraining effects on complaints related to the menopausal transition. To estimate the onset of significant detraining effects on menopausal symptoms and to derive recommendation for intended training breaks, future studies should determine detraining periods shorter than 3 months. Additionally, it is important to verify the results of the present study for the highly relevant cohort of perimenopausal women.

## Data Availability

The raw data supporting the conclusions of this article will be made available by the authors, without undue reservation.
